# Massive Benign Pneumatosis Intestinalis

**DOI:** 10.5334/jbsr.2188

**Published:** 2020-11-24

**Authors:** Louis Vanderschueren, Bruno Coulier

**Affiliations:** 1 Clinique St Luc, BE; 2 Clinique Saint-Luc, Bouge, BE

**Keywords:** Pneumatosis intestinalis, cancer, 5-Fluorouracil, colon, computed tomography

## Abstract

**Teaching point**: CT may help distinguishing benign from life-threatening pneumatosis intestinalis.

## Case report

A 78-year old woman presented with epigastric pain and non-bloody diarrhea 6 days after a second cure of adjuvant chemotherapy for gastric adenocarcinoma (the FOLFIRI protocol that includes Folinic acid, 5-Fluorouracil (5-FU) and Irinotecan).

**Figure 1:**
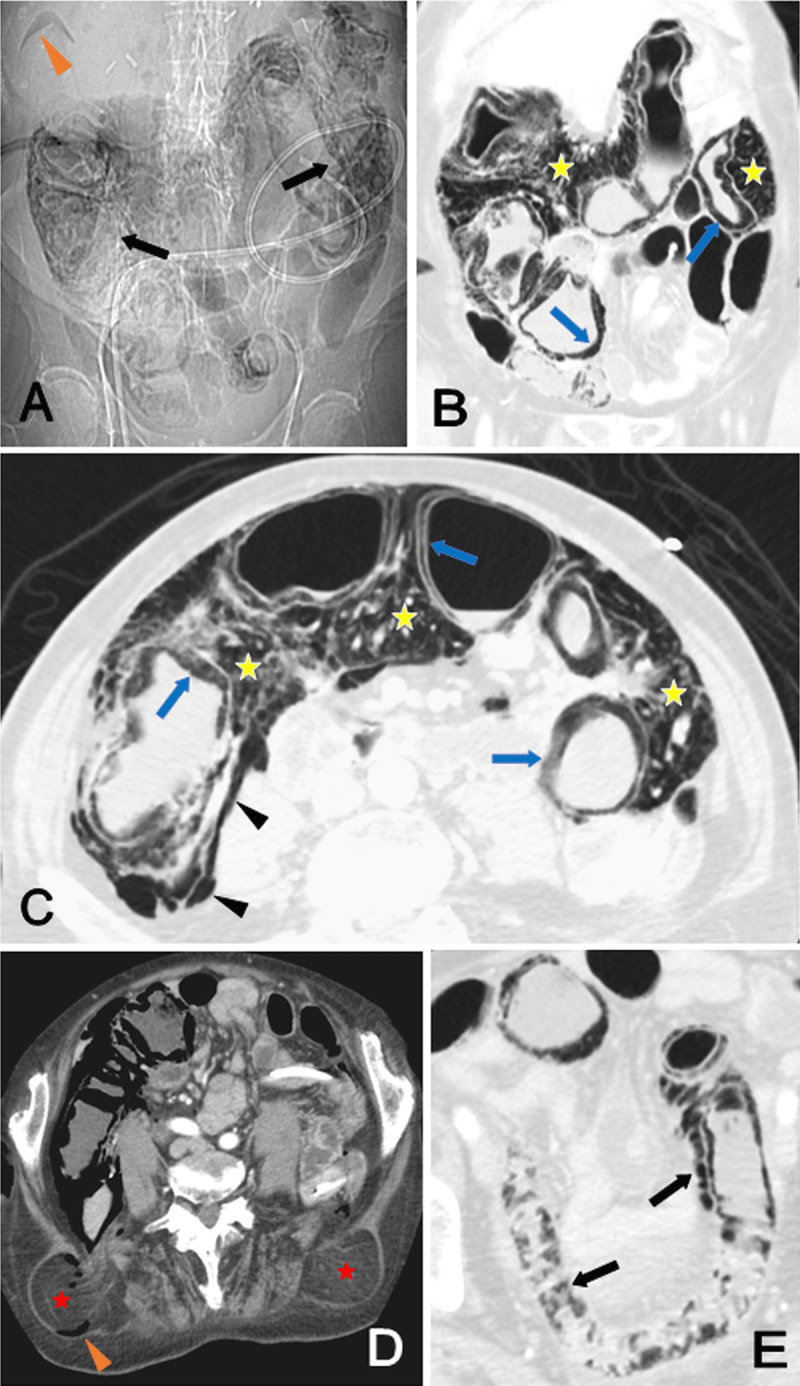


Massive Pneumatosis Intestinalis (PI) could be seen (Figure 1, A to E) on the scout view (black arrows on A). On subsequent coronal (B) and axial (C to E) Computed Tomography (CT), colonic submucosal (blue arrows), extensive subserosal, mesenteric (yellow stars) and free posterior retroperitoneal gas (black arrowheads) were depicted. Gas were also found within in a right Grynfelt hernia (orange arrowhead on A, red stars on D). The distal ileum was slightly affected (black arrows on E). Pneumoperitoneum, portal gas, bowel wall thickening, mesenteric fat stranding and ascites were absent. The precise etiology of PI could not be clearly identified, even though contribution of chemotherapy (notably 5-FU) was likely. Given the absence of alarm CT findings, the patient was successfully treated conservatively.

## Comment

Pneumatosis intestinalis which is defined as the presence of gas within the digestive tract wall is a radiographic finding resulting of a large spectrum of underlying diseases. The nature and severity of the disease determines the clinical significance of PI, not the amount of air. Initially considered indicative of advanced intestinal infarction, PI may be found in benign conditions such as immunosuppressive states.

Pathogenesis of PI is multifactorial but two main theories emerge. The ‘bacterial theory’ hypothesizes the action of gas-forming bacilli entering the submucosa through mucosal breaks or increased mucosal permeability. The ‘mechanical theory’ hypothesizes that increasing of intraluminal bowel pressure with loss of intestinal mucosal integrity allows normal bowel gas to dissect the bowel wall.

Regarding this, patients with cancer have a greater propensity to develop PI - often benign - because they cumulate multiple risk factors (surgery, medical procedures and immunosuppression). Thrombogenic and vasospastic effects of 5-FU on the intestinal epithelium may have played a role in our patient.

Independently from the clinical status, CT may help differentiate benign from worrisome PI and guide proper clinical treatment [1]. Alarm signs include porto-mesenteric gas, mesenteric stranding, bowel dilatation and thickening, ascites, and confinement of PI to the small bowel. Colonic PI appears more frequently benign. Pneumoperitoneum may be seen in benign PI.
